# Norwegian reference data on the Fatigue Questionnaire and the Patient Health Questionnaire-9 and their interrelationship

**DOI:** 10.1186/s12991-020-00311-5

**Published:** 2020-10-09

**Authors:** Alv A. Dahl, Kjersti Støen Grotmol, Marianne Jensen Hjermstad, Cecilie Essholt Kiserud, Jon Håvard Loge

**Affiliations:** 1 National Advisory Unit On Late Effects After Cancer Treatment, Oslo University Hospital, 0424 Oslo, Norway; 2 Faculty of Medicine, University of Oslo, 0316 Oslo, Norway; 3 European Palliative Care Research Centre (PRC), Department of Oncology, Oslo University Hospital, and Institute of Clinical Medicine, University of Oslo, Oslo, Norway; 4 Regional Advisory Unit for Palliative Care, Department of Oncology, Oslo University Hospital, 0424 Oslo, Norway

**Keywords:** Reference data, The Fatigue Questionnaire, The Patient Health Questionnaire, Major depressive episode, Chronic fatigue

## Abstract

**Background:**

Population-based reference data on frequently used questionnaires are important for comparative purposes. Due to changes in health and lifestyles, such data should be updated every other decade. The objectives of this study were to establish Norwegian population-based reference data on the Fatigue Questionnaire (FQ) and the Patient Health Questionnaire-9 (PHQ-9) on depression, to compare the FQ-scores with our previous reference data from 1996, and to explore the relationship between the scores on these two instruments.

**Methods:**

In 2015, a representative sample of 6,012 Norwegians aged 18–80 years was mailed a questionnaire including the FQ and the PHQ-9, and 36% responded. Complete FQ-scores were delivered by 2,041 subjects, and complete PHQ-9 scores by 2,086 subjects. The scores are displayed according to sex and 10-year age groups.

**Results:**

Few 2015 mean scores of mental, physical, and total fatigue differed significantly from those of 1996, and the same was found for the prevalence rates of chronic fatigue. The exception was a significantly lower prevalence in 2015 of mean fatigue scores and prevalence of chronic fatigue in females ≥ 60 years. The prevalence of major depressive episode (MDE) based on the PHQ-9 sum score cut-off ≥ 10 was 5.9% for males and 9.8% for females, and 2.5% and 3.8% using a DSM-based algorithm with at least five endorsed criteria including either anhedonia or depressed mood. The correlation between the FQ and the PHQ-9 was 0.59, implying 36% shared variance.

**Conclusions:**

This study showed considerable interrelationship between the FQ and the PHQ-9 constructs. The reference data show that scores on the FQ have only improved significantly in persons aged 60 or more years between 1996 and 2015. Our prevalence findings of MDE based on the PHQ-9 are in accordance with the findings from other countries. The FQ and the PHQ-9 should be used together in epidemiological and clinical studies.

## Background

According to the Merriam-Webster Dictionary, depression is a state of feeling sad, and fatigue is a state of weariness or exhaustion [1]. Both states are described both as symptoms and as clinical syndromes. Conceptually, the two states overlap partly because fatigue is a symptom of the depressive syndrome, and their interrelationship is, therefore, of considerable clinical relevance, but rarely investigated. Major depressive episode (MDE) in the DSM-5 [2] and Depressive episodes (F32) in the ICD-10 [3] are defined as mental disorders, and both have fatigue or loss of energy as diagnostic criterion [2] or a clinical guideline item [3], respectively. Fatigue is a complex and unspecific symptom observed in several somatic diseases and mental disorders. Fatigue is a central symptom of both MDE and Depressive episodes (as well as) and of ICD-10 F48.0 Neurasthenia (not included in the DSM-5), as well of unclassified syndromes such as myalgic encephalopathy (ME) and chronic fatigue syndrome (CFS) [4].

Depressive and fatigue syndromes are common, and of major clinical interest in both primary care and hospital settings. Assessment of these syndromes is primarily based upon patients’ reports, and several patient-reported outcome measures (PROMs) have been developed. To separate the two syndromes is also of considerable clinical interest since depression is quite amenable to treatment [5] and carries an increased risk of suicide that is not found for the fatigue syndrome, which is less amenable to treatment [6].

A Norwegian population-based study found a point prevalence of 11.4% for fatigue lasting for six months or more defined as chronic fatigue, based on self-report with the Fatigue Questionnaire [7]. Based on a structured interview, the population-based 12-month prevalence of DSM-III-R MDE was 7.3% in the capital of Oslo [8] and 3.7% in the rural Sogn and Fjordane county [9]. To our knowledge, there are no Norwegian studies of the overlap between fatigue and depression. However, many studies have shown a high co-occurrence of fatigue and depression in the general population, and increased risk for fatigue in persons with depression and vice versa [10, 11]. All these studies showed a higher prevalence in females compared to males.

Use of national population-based reference data for comparative purposes has become a widely used strategy for interpreting PROMs in diseased populations anchoring the results to such data. This provides the opportunity to assess the clinical significance of results adjusting for sex and age as well as other relevant characteristics.

The Wessely group in London developed the Fatigue Questionnaire (FQ), and the 11 fatigue items are shown in Table 1. The development was originally based upon a need for standardized assessment of fatigue as part of the CFS. The authors recognized a need for a standardized assessment of fatigue per se, so all items specific for the CFS were removed [12]. The FQ has become widely used because of its coverage of the most relevant physical and mental fatigue symptoms and the instrument has demonstrated good psychometric properties. In Norway, the FQ has been commonly used in various disease groups since late 1990s. Population-based reference data from 1996 have been published [7] and used for comparative purposes in several publications from various diseased samples [13–15].

**Table 1:** The symptom items of the FQ and the PHQ-9 scales

FQ items	PHQ-9 items
*1. Problems with tiredness*	1. Little interest or pleasure in doing things
2. Needs more rest	2. Feeling down, depressed, or hopeless
*3. Feels sleepy or drowsy*	3. Trouble falling or staying asleep
4. Problems starting things	4. Feeling tired or having little energy*
*5. Lacking energy*	5. Poor appetite or overeating
6. Less strength in muscles	6. Feeling bad about yourself
7. Feels weak	7. Trouble concentrating*
*8. Difficulty concentrating*	8. Changed psycho-motoric tempo
9. Makes slips of tongue when speaking	9. Better off dead or hurting oneself
10. More difficult to find the correct word	
11. Memory problems	

^*^ In *italics* fatigue items common for the FQ and the PHQ-9. The FQ items #1 and #5 cover the PHQ-9 item #4, the FQ item #8 covers the PHQ-9 item #7

The Spitzer group in New York developed the Patient Health Questionnaire-9 (PHQ-9) as part of their project of making diagnoses of mental disorders more feasible in primary care [16, 17]. They gave the nine items under criterion A of the MDE criteria of the DSM-IV [18] a self-rating format (Table 1). Originally developed for screening of depression in primary care, the PHQ-9 also has also become widely used in hospital settings. Several countries like Germany, Sweden, and South Korea have published reference data [19–21]. The PHQ-9 has only recently been translated into Norwegian, and no Norwegian reference data have been published so far.

Both the FQ and the PHQ-9 were included in a recent representative Norwegian population survey collecting data on various PROMs [22]. Data from this survey allowed us to: (1) Report 2015 reference data for the FQ and the PHQ-9 according to sex and age groups; (2) Compare the FQ reference scores from 2015 to those of 1996, and (3) Explore the relationship between the FQ and the PHQ-9 scores.

## Methods

### Samples

#### Reference data of 1996

A representative sample of the general Norwegian population by sex and place of residence, aged 19–80 years, was drawn at random. Among them 3,452 subjects were eligible for the study, and 2,323 returned questionnaires (response rate 67%). Of these respondents 2,287 delivered complete FQ forms [7].

#### Reference data of 2015

The Bring Dialog Company randomly drew 6,012 subjects, aged 18–80 years, and representative of the general Norwegian population with respect to age, sex and place of residence [22]. They received a mailed questionnaire packet including the FQ, the PHQ-9, and two other PROMs plus supplementary questions concerning socio-demographics, lifestyle, etc. Of the 2,142 returned questionnaires (response rate 36%), 46 were omitted (24 blank and 22 without sex) leaving 2,096 respondents. Among them 2,041 (97.4%) respondents delivered complete FQ forms and 2,086 (99.5%) complete PHQ-9 forms. Valid forms on both instruments were delivered by 2,037 respondents.

The demographics of the 1996 and 2015 FQ samples are displayed in Table 2.

**Table 2:** Comparison of the 1996 and 2015 FQ samples

Variables	1996 sample *(N* = *2,323)*	2015 sample *(N* = *2,041)*	*p* value
Age (years), mean (SD)	44.9 (16.5)	55.5 (14.1)	< *0.001*
Age groups (years), *N* (%)			< *0.001*
< 29	510 (22)	101 (5)	
30–39	487 (21)	196 (10)	
40–49	446 (19)	392 (19)	
50–59	363 (16)	466 (23)	
≥ 60	517 (22)	886 (43)	
Sex, *N* (%)			*0.02*
Female	1,192 (51)	1,118 (55)	
Male	1,131 (49)	923 (45)	
Educational status, *N* (%)			< *0.001*
Second level, first stage (lower)	621 (27)	281 (14)	
Second level, first stage (medium)	1,036 (45)	831 (41)	
Third level (university)	643 (28)	919 (45)	
Married/cohabitant	1,609 (70)	1,549 (76)	< *0.001*

### The FQ and the PHQ-9

The *FQ* contains 11 fatigue-related symptoms experienced during the last month compared with how the subject felt when he/she last was feeling well. Four items concern mental and seven physical fatigue (Table 1). Each item is rated from 0 (‘less’/’better than usual’) to 3 (‘much worse than usual’). The mental fatigue sum score ranges from 0 to 12 and the physical fatigue sum score from 0 to 21, and higher scores mean more fatigue on both subscales. The total fatigue sum score represents the sum of all 11 items and ranges from 0 to 33. An additional FQ item covers the duration of the fatigue experience with one response alternative being “6 months or more”.

To identify cases with chronic fatigue (CF), a dichotomized score for each response alternative (0 = 0, 1 = 0, 2 = 1, 3 = 1) was used with a range of 0 to 11, and CF was defined as a dichotomized sum score of ≥ 4 with a duration of ≥ 6 months [23].

The *PHQ-9* covers depressive symptoms experienced during the last 2 weeks, and each item is scored from 0 (‘not at all’) to 3 (‘nearly every day’), providing a 0–27 severity score.[16]. MDE is defined either by a sum score ≥ 10 [17], or by an algorithm as in the DSM system. We used the algorithm recommended by the Spitzer group in which an item is counted as a positive criterion if scored above threshold. Items #1–8 were scored as ‘bothersome’ for at least ‘more than half the days’ (score 2), or ‘on some days’ (score 1) on item #9 (thoughts about suicide or self-harm), to fulfill the algorithm. At least five criteria must be present among which either item #1 (anhedonia) or item #2 (depressed mood) is obligatory [17, 24, 25].

### Data analysis

Descriptive statistics were applied (concerning) of means and proportions, which presented with their 95% confidence intervals (95%CI). Non-overlapping 95%CI represent a *p* value of < 0.05 [26]. The associations between the FQ and the PHQ-9 items and sum scores were examined with Spearman’s correlation coefficient *r*. Correlations were also reported as shared variance defined as the correlation coefficient squared in percent. Internal consistencies of the instruments were tested with Cronbach’s coefficient alpha. The *p* value was set at < 0.05, and all tests were two-sided. The statistical software applied was IBM SPSS version 25 for PC (IBM Corporation, Armonk, USA).

## Results

### General findings of the 2015 sample

Attrition analysis of non-responders versus responders of 2015 sample has already been published [22]. Significantly more women than men responded to the invitation, and the responders were significantly older than the non-responders. The 2015 sample consisted of 923 (45%) males and 1,118 (55%) females who completed the FQ, and of 943 (45%) men and 1,143 (55%) women who completed the PHQ-9, respectively.

### Findings on the FQ in 1996 and 2015

Internal consistencies of the FQ measured by Cronbach’s coefficient alphas were 0.88 for physical, 0.74 for mental, and 0.88 for total fatigue in the 2015 sample. Concerning the FQ samples of 1996 and 2015, several significant differences were observed on socio-demographic variables (Table 2).

The prevalence rates of CF according to sex and age groups are displayed in Table 3. No significant differences of prevalence rates were observed for males in any age groups. For females, however, the prevalence rates in 2015 are significantly higher in the 18–29 years age group and significantly lower in the ≥ 60 years age group.

**Table 3:** Prevalence of chronic fatigue (95% confidence intervals) according to sex and 10-year age group I 1996 and 2015

Sex and age groups (N = 1996/2015)	Chronic fatigue 1996	Chronic fatigue 2015
	% (95%CI)	% (95%CI)
Males		
18–29 years (*N* = 233/36)	5.2 (2.9–8.9)	13.9 (5.6–29.1)
30–39 years (*N* = 245/80)	6.9 (4.3–10.9)	10.0 (4.9–18.8)
40–49 years (*N* = 216/165)	9.7 (6.4–14.5)	15.2 (10.4–21.5)
50–59 years (*N* = 180/209)	12.2 (8.2–17.9)	11.5 (7.8–16.6)
> 60 years (*N* = 238/433)	16.8 (12.6–22.1)	12.7 (9.9–16.2)
Total	10.1 (8.4–12.0)	12.7 (10.7–15.0)
Females		
18–29 years (N = 272/65)	4.8 (2.7–8.1)*	**23.1 (14.4–34.8)**
30–39 years (N = 235/116)	11.9 (8.3–16.7)	13.8 (8.6–21.3)
40—–9 years (N = 224/227)	13.8 (9.9–19.0)	15.0 (10.9–20.2)
50–59 years (N = 181/257)	10.5 (6.8–15.9)	15.2 (11.3–20.1)
> 60 years (N = 263/453)	21.7 (17.1–27.1)*	**11.3 (8.7–14.5)**
Total	12.6 (10.8–14.6)	13.9 (12.0–16.0)

^*^Significant differences between the 1996 and 2015 samples are set in bold types

As to the FQ mean scores, the mental and total scores for males ≥ 60 years were significantly lower in 2015 compared to 1996. The significant age group differences observed in 1996 were not the same in 2015. The only significant mean difference was a lower mean score for total fatigue in the 30–39 years versus ≥ 60 years age group (Table 4). In females, the age group of ≥ 60 years reported significantly lower mean scores for all FQ dimensions in 2015. The significant age group differences observed for physical and total fatigue in 1996 were not found in 2015.

**Table 4:** Physical, mental, and total fatigue mean scores (with 95% confidence intervals) in 1996 and 2015 according to sex and 10-year age groups

Sex and age groups (N = 1996/2015)	Physical 1996	Physical 2015	Mental 1996	Mental 2015	Total 1996	Total 2015
	Mean (CI)	Mean (CI)	Mean (CI)	Mean (CI)	Mean (CI)	Mean (CI)
Males						
18–29 years (*N* = 233/36)	6.9 (6.6–7.2)	7.3 (6.2–8.5)	4.2 (4.0–4.4)	4.2 (3.6–4.8)	11.1 (10.6–11.6)	11.6 (10.0–13.2)
30–39 years (*N* = 245/80)	7.3 (7.0–7.6)	7.7 (6.5–8.1)	4.2 (4.0–4.4)	4.1 (3.8–4.4)	11.5 (11.0–12.0)	10.7 (9.8–11.6)
40–49 years (*N* = 216/165)	7.4 (7.0–7.8)	7.1 (6.6–7.6)	4.3 (4.1–4.5)	4.1 (3.9–4.3)	11.7 (11.2–12.2)	11.7 (11.0–12.4)
50–59 years (*N* = 180/209)	8.1 (7.6–8.6)	7.7 (7.3–8.1)	4.5 (4.3–4.7)	4.1 (3.9–4.3)	12.6 (12.0–13.2)	11.7 (11.2–12.2)
≥ 60 years (N = 238/433)	8.4 (8.0–8.8)	7.8 (7.5–8.1)	4.5 (4.3–4.7)*	**4.1 (4.0–4.2)**	12.9 (12.4–13.4)*	**12.0 (11.7–12.3)**
Females						
18–29 years (N = 272/65)	8.0 (7.6–8.4)	8.9 (8.0–9.8)	4.4 (4.2–4.6)	4.3 (3.8–4.8)	12.3 (11.8–12.8)	13.3 (12.1–14.5)
30–39 years (N = 235/116)	8.2 (7.8–8.6)	8.0 (7.3–8.7)	4.3 (4.1–4.5)	4.3 (4.0–4.6)	12.5 (12.0–13.0)	12.3 (11.3–13.3)
40–49 years (N = 224/227)	7.9 (7.5–8.3)	8.2 (7.7–8.7)	4.3 (4.1–4.5)	4.3 (4.1–4.5)	12.2 (11.6–12.8)	12.5 (11.8–13.2)
50–59 years (N = 181/257)	7.8 (7.4–8.2)	8.1 (7.7–8.5)	4.3 (4.1–4.5)	4.3 (4.1–4.5)	12.2 (11.7–12.7)	12.4 (11.9–12.9)
≥ 60 years (N = 263/453)	9.0 (8.6–9.4)*	8.0 (7.7–8.3)*	4.4 (4.2–4.6)*	**4.0 (3.9–4.1)**	13.4 (12.9–13.9)*	**12.0 (11.6–12.4)**

* Significant differences in mean scores between the 1996 and 2015 samples are set in bold types

### PHQ-9 findings of 2015

The Cronbach’s coefficient alpha was 0.87 for the PHQ-9. The mean PHQ-9 scores according to the cut-off score and the algorithm score are displayed in Table 5 divided by sex and age groups. For males, no significant differences in mean scores were observed between the 10-year age groups. In contrast, among females the mean PHQ-9 scores in the 18–29 years age group were significantly higher relative to the age groups ≥ 50 years and to the overall mean score.

**Table 5:** PHQ-9 defined prevalence rates (%) (95%Confidence intervals) of major depressive episodes (MDE) and mean scores (95%confidence intervals) according to sex and age groups

Males	18–29 years *(N* = *36)*	30–39 years *(N* = *81)*	40–49 years *(N* = *167)*	50–59 years *(N* = *216)*	60–69 years *(N* = *256)*	70–79 years *(N* = *187)*	Total *(N* = *943)*
MDE cut-off ≥ 10*	11.1 (3.8–25.9)	7.4 (3.2–15.5)	8.3 (5.0–13.7)	6.0 (3.5–10.1)	4.7 (2.6–8.1)	3.7 (1.7–7.7)	5.9 (4.6–7.6)
MDE algorithm*	2.8 (0.0–15.4)	3.7 (0.8–10.8)	3.6 (1.5–7.8)	2.8 (1.1–6.1)	2.0 (0.7–4.6)	1.6 (0.3–4.8)	2.6 (1.7–3.8)
* PHQ-9 mean score#*	*3.8 (2.4–5.2)*	*3.4 (2.4–4.0)*	*3.2 (2.5–3.9)*	*2.7 (2.2–3.2)*	*2.5 (2.1–2.9)*	*2.4 (1.9–2.7)*	*2.7 (2.5–2.9)*
Females	18–29 years *(N* = *68)*	30–39 years *(N* = *120)*	40–49 years *(N* = *231)*	50–59 years *(N* = *259)*	60–69 years *(N* = *260)*	70–79 years *(N* = *205)*	Total *(N* = *1143)*
MDE cut-off ≥ 10*	17.6 (10.2–28.5)*	10.8 (6.3–17.8)	13.4 (9.6–18.5)	9.3 (6.3–13.5)	7.7 (5.0–11.6)	5.9 (3.3–10.1)*	9.8 (8.2–11.7)
MDE algorithm*	8.8 (3.8–18.3)*	7.5 (3.8–13.8)	4.3 (2.3–7.9)	2.7 (1.2–5.6)	3.4 (1.7–6.5)	1.0 (0.0–3.7)*	3.8 (2.8–5.0)
* PHQ-9 mean score#*	***5.2 (4.0–6.4)#***	*4.1 (3.1–5.1)*	*3.8 (3.2–4.4)*	***3.4 (2.9–3.9)#***	***3.0 (2.5–3.5)#***	***2.9 (2.4–3.4)#***	***3.5 (3.3–3.7)#***

* Significant age group prevalence differences in bold. #Significant age group mean differences in bold and italics

Based on the PHQ-9 sum score ≥ 10 definition of MDE, the prevalence was 8.1% (95%CI 6.9—9.2%) in the total sample, and among males 5.9% (95%CI 4.4–7.4%) and females 9.8% (95%CI 8.2–11.7%). Sex and age group distributions are shown in Table 5, and significant differences were observed between the 18–29 years versus the 70–79 years age groups for females, while no significant age differences were observed for males.

According to the Spitzer group’s algorithm, cases of MDE were 3.2% (95%CI 2.4–4.0%) in the total sample, and among males 2.6% (95%CI 1.7–3.8%) and females 3.8% (95%CI 2.8–5.0%). No significant age group differences were observed for males, but significant differences were observed between the 18–29 years and the 70–79 years age groups for females (Table 5).

Sixty-seven persons (3.2% of the sample) were defined as depressed by both definitions, while 101 (4.8%) fulfilled the dimensional definition only, and no person fulfilled the algorithm definition only. The prevalence of MDE defined by the PHQ-9 sum score was significantly higher for all age groups and for both sexes compared to MDE defined by the algorithm. The differences between the MDE definitions were significant for both males’ and females’ total prevalence, and for the prevalence rates of females age 40–49, 50–59, and 70–79 years.

For the PHQ-9 mean scores, no significant differences were observed between the age groups in males, while among females the mean score of the 18–29 years age group was significantly higher than for the age groups of 50–59 years and older (Table 5).

### Relationship between the FQ and the PHQ-9

According to Table 1, the FQ has two items that correspond with three PHQ-9 items: FQ item #4 covers PHQ-9 items #1 and #5, FQ item #7 and PHQ-9 item #8 seem equal, while FQ item #3 and PHQ-9 item #3 are somewhat different concerning sleep problems.

The correlation matrix (Table 6) showed that most correlation coefficients were < 0.50, implying less than 25% shared variance. Only PHQ-9 item #4 (feeling of tiredness) showed four correlations between 0.52 and 0.61 with the FQ items #1 (tiredness), #2 (need more rest), #3 (feels sleepy and drowsy), and #5 (lacking energy) as well as the total fatigue score. Maximum shared variance between the item sets was 37% for FQ item #5 and PHQ item #4. The FQ items #1, 2, 4 and 5 showed correlations of 0.50 to 0.61 with the PHQ-9 sum score. The FQ and the PHQ-9 sum scores showed a correlation of r = 0.59, implying 35% shared variance.

**Table 6:** Correlation coefficients of the 11 FQ items versus the 9 PHQ-9 items correlations with the FQ and PHQ-9 sum scores (N = 2,037)

	FQ1	FQ2	FQ3	FQ4	FQ5	FQ6	FQ8	FQ9	FQ10	FQ11	FQ
PHQ1	0.42	0.40	0.37	0.44	0.49	0.25	0.35	0.13	0.24	0.30	0.48
PHQ2	0.38	0.38	0.34	0.37	0.46	0.21	0.32	0.09	0.19	0.30	0.43
PHQ3	0.33	0.34	0.30	0.30	0.38	0.27	0.24	0.09	0.19	0.20	0.40
PHQ4	**0.52**	**0.54**	**0.52**	0.45	**0.61**	0.33	0.34	0.09	0.21	0.30	**0.59**
PHQ5	0.30	0.35	0.30	0.33	0.38	0.23	0.27	0.10	0.17	0.24	0.38
PHQ6	0.33	0.32	0.32	0.34	0.20	0.42	0.25	0.11	0.20	0.28	0.40
PHQ7	0.27	0.31	0.29	0.29	0.34	0.20	0.40	0.14	0.28	0.37	0.37
PHQ8	0.22	0.23	0.22	0.25	0.25	0.15	0.20	0.14	0.15	0.20	0.30
PHQ-9	0.21	0.23	0.22	0.25	0.25	0.15	0.20	0.14	0.15	0.20	0.27
PHQ	**0.50**	**0.52**	0.48	**0.51**	**0.61**	0.35	0.46	0.19	0.30	0.37	**0.59**

**Bold numbers** showing correlations with Spearman’s coefficients r ≥ 0.50

## Discussion

### Main findings

Males and females ≥ 60 years had significantly lower mean scores of mental and total fatigue in 2015 compared to 1996, and for the females that was the case also for physical fatigue. No other significant differences between age groups on FQ mean scores were observed over time. Concerning prevalence of CF, only females ≥ 60 years showed a reduction from 1996 to 2015. The increase in CF among females aged 18–29 we consider an artifact (see below). The same was relevant for the high PHQ-9 mean score and prevalence of MDE in that group. The prevalence rates of MDE based on PHQ-9 sum scores were higher than those based on the algorithm, but no significant differences were found between age groups in males, or between the age groups over 30 years in females. The shared variance between the FQ and the PHQ scales was 35%.

### Representativity of the 2015 sample

The randomly drawn national 2015 sample was representative of the Norwegian population concerning age, sex and place of dwelling. However, the response rate was only 36%. The previous attrition analysis showed that significantly more women than men responded, and the participants were significantly older than the non-participants [22]. About 21% of the population was between 18 and 29 years, but this age group only represented 5% of our sample. Hence, the number of participants in this age group was very low; only 36 males responding to the FQ and the PHQ-9, and 65 and 68 females, respectively. The significantly higher rates of males and females with CF in 2015 in this age group, indicate a bias towards more ill responders. Thus, we suggest that our FQ and PHQ-9 results for the 18–29 age group, should not be used as reference data.

In contrast, individuals aged 67 years or above were over-represented since they constituted 27% of the sample, while only representing 18% of the population. The high proportion of respondents over 70 years may reflect the increased life expectancy in Norway, and active and engaged persons constitute a larger proportion of this age group. Since fatigue and depression are characterized by lack of energy and initiative, which increases the risk of non-participation, our sample also carries a risk of under-reporting for this reason. We are not able to estimate of that factor on our findings.

### Decline in response rate

The decline in response rate from 67% in 1996 to 36% in 2015 is in accordance with both national [27] and international trends [28] concerning mailed questionnaires. Several factors may be operating, but in general, the population has become more restrictive in sharing personal information. Another factor is that the general population nowadays is exposed to a higher rate of mailed and electronic questionnaires than previously, not only from the field of medicine.

In 2018, 96% of all Norwegian households with at least one person < 75 years had access to the Internet, and an alternative had been to make a digital rather than postal survey. However, that approach could trigger still more fear of spreading of sensitive information, and the response rate is not necessarily higher than in paper-based surveys [29]. Web-based studies, therefore, do not solve the problem of sinking response rate to population-based health studies in Norway.

### Findings of the PHQ-9

The first Norwegian reference data on the PHQ-9 showed lower mean PHQ-9 scores with increasing age for both sexes, and consistently higher mean scores in females for all age groups. The sex differences of the mean scores of the PHQ-9 were also observed in a German population based study [30], but for both sexes, they observed an increase in mean PHQ-9 scores with age. The reason for the differences may be that their study had better representativity with 63% response rate.

In our study, closely the same pattern was observed for MDE defined by cut-off ≥ 10 with reduced prevalence with increasing age and female sex, but we did not find any other papers with such PHQ-9 population data.

MDE defined by the algorithm score showed lower prevalence rates than defined by cut-off score for all age groups and both sexes. The discrepancy of these prevalence rates of MDE presents a problem for the use of the PHQ-9 as a clinical screening instrument for depression. Especially since the discrepancy of prevalence rates between the dimensional and algorithmic definitions of MDE also was observed in studies from other countries [20213031] [20, 21, 30, 31].

For PHQ-9 score ≥ 10 definitions of MDE, we observed no significant prevalence differences in males between our study and the others; while for females, our study had significantly higher prevalence than reported by Kocalevent et al. [31]. Concerning total prevalence rates, our study found significantly higher rates than both Shin et al. [21] and Kocalevent et al. [31]. Based on the MDE algorithm, our prevalence rates did not differ significantly from the other studies concerning sex-based and total prevalence rates.

### Fatigue changes over time

Several demographic and lifestyle-related changes have occurred in Norway during the last decades. Mean life expectancy has increased for both sexes to 81 years for males and 84 years for females in 2016. Self-reported health and activities of daily living have improved in persons above 70 years over the last two decades [32]. Of today’s adult Norwegian population, 13% are daily smokers, obesity (BMI ≥ 30) 12, 32% consume alcohol once a week or more frequently, and 71% are physically active once a week or more [33]. Fourteen per cent of the population are immigrants, making the Norwegian population more diverse than before. In addition, there are significant differences concerning socio-demographic variables in our 1996 and 2015 samples as shown in Table 2. In spite of these social changes and sample differences, the levels of physical, mental and total fatigue show only significant changes in the ≥ 60 year age group for both sexes. We consider the reduction of fatigue among elderly persons to be a consequence of their improved health status [32].

The consistent increase in physical fatigue with age in males observed in 1996 [7] was not observed in 2015. It is tempting to speculate that this finding could in part be explained by improved health in middle-aged men [32].

Concerning the prevalence of CF, no significant changes were observed for males, but we observed a significant reduction among women ≥ 60 years, probably due to improved health in this group of women. The significant increase in the prevalence of CF among women aged 18–29 years, we consider due to sampling bias, and we remind about our concern to not use data from that age group as reference data for either sex.

### The relationship between the FQ and the PHQ-9

As indicated in Table 1, three items concerning energy, tiredness, and concentration are common to both instruments. The correlation matrix between the FQ and the PHQ-9 (Table 6) mostly showed coefficients below 0.50, except for the PHQ-9 items #4 ‘Feeling tired or having little energy’ that had coefficients above 0.50 with four physical fatigue items (#1, 2, 3, and 5) related to tiredness, lack of energy, and need for rest. The 35% shared variance indicate that these PROMs could be used together, since 65% of their variance is separate.

### Mechanisms of fatigue and depression

Our findings have confirmed the close relationship between fatigue and depression. Several explanations for this fact have been proposed. One of them is heredity, as documented in the study of a twin sample. Multivariate twin modeling estimated a common additive genetic component which explained 25%, and 20% of the variance in depression and fatigue, respectively. For depression, environmental factors explained 28% and for fatigue 54% of the variance [34].

Another explanation concerns malfunctioning neural circuits and neurotransmitters [10]. They build on the observation that treatment with antidepressants often is effective on the affective component of MDE, but less so on the lack of energy (fatigue) component. Therefore, antidepressants are considered to have different effects on the depression and fatigue neural circuits that may be due to different neurotransmitter profiles.

These and other potential explanations need further research.

### Strength and limitations

We have produced Norwegian normative data for the FQ and the PHQ-9 scales. The FQ results show reduced fatigue in persons ≥ 60 years from 1996 to 2015 possibly reflecting improved general health in Norwegian during that period. The PHQ-9 data are new, but in line with the findings of other national samples [20, 21, 30, 31].

Limitations are the response rate of 36% leading to low numbers of responders in the 18–29 years age group; thus, the FQ and PHQ-9 data reported by that age group should be considered critically.

## Conclusion

Although the FQ and the PHQ-9 showed considerable item overlap and their sum scores had 35% shared variance, 65% of the variance is not shared. Our findings, therefore, indicate that these PROMs could be used together supplementing each other.

## Data Availability

The data file of the study is located at the research server of Oslo University Hospital, and all authors have access to that file. According to Norwegian data regulations, the authors are not allowed to export that datafile elsewhere and thereby making it publicly available.

## Abbreviations

95%CI: 95% Confidence Interval
CFS: Chronic Fatigue Syndrome
DSM-IV: Diagnostic and Statistical Manual, 4th edition
DSM-5: Diagnostic and Statistical Manual, 5th edition
FQ: Fatigue Questionnaire
MDE: Major Depressive Episode
ME: Myalgic encephalopathy
PHQ-9: Patient Health Questionnaire
PROMs: Patient-rated Outcome Measures
